# Meiotic Recombination May Be Initiated by Copy Choice During DNA Synthesis Rather than Break/Join Mechanism

**DOI:** 10.3390/ijms26199464

**Published:** 2025-09-27

**Authors:** Lei Jia, Na Yin, Xiaolin Wang, Jingyun Li, Lin Li

**Affiliations:** 1 State Key Laboratory of Pathogen and Biosecurity, Academy of Military Medical Sciences, Beijing 100850, China; yinna15866875835@163.com (N.Y.); woodsxl@163.com (X.W.); lijy@bmi.ac.cn (J.L.); 2 Key Laboratory for the Prevention and Control of Emerging Infectious Diseases and Biosafety, Department of Microbiological Laboratory Technology, School of Public Health, Cheeloo College of Medicine, Shandong University, Jinan 250012, China

**Keywords:** DNA meiotic recombination, meiosis, chromosome, branched structure, copy choice, resolution pattern of a Holliday Junction, genetic

## Abstract

Our understanding of the molecular mechanisms by which DNA meiotic recombination occurs has significantly increased in the past decades. A more representative molecular model has also undergone repeated revisions and upgrades with the continuous expansion of experimental data. Considering several apparent issues in the field, we intend to make necessary upgrades to previous models and reanalyze those data, exploring structural details and molecular mechanisms of DNA meiotic recombination. Eligible studies were identified from PubMed/Medline (up to June 2024). Key related publications and experimental data were retrieved from eligible studies, displaying five major issues. Meanwhile, the biophysical modeling method was used to establish an enlacement model. Then, the model was used to wholly reanalyze the collected data. An updated molecular model was supplemented. In the current model, a copy choice mechanism can initiate DNA meiotic recombination. The copy choice is based on a branched structure of DNA, which results from relative motion between homologous single strands. The reanalysis of previous experimental data based on this model can lead to new interpretations that can better address the discrepancies between previous experimental observations and theoretical models, including (1) the intertwinement model having embodied the particular characteristics of the SDSA model; (2) hDNA arising from JM resolution rather than being followed by a JM; (3) strand specificity of hDNA mismatch repair seeming to be an illusion and copy choice more likely to be the actual state; (4) parity in resolution patterns of a dHJ leading to parity of gene conversion; (5) the cooperation of multiple HJs readily generating a high correlation between gene conversion and crossover; and (6) transpositional recombination and site-specific recombination seeming to have a common pathway to meiotic recombination. The results indicate that both revisions and reanalysis are necessary. The novel interpretations would be critical to the understanding of the mechanisms of DNA recombination as well as its role in DNA repair. Additionally, the work could have implications for how the field views the importance of factors such as Spo11 or the mechanisms that drive meiotic pairing.

## 1. Introduction

Types of DNA recombination include homologous recombination (HR) and nonhomologous recombination (non-HR) (transpositional recombination and site-specific recombination). Considering that fully documented accounts for DNA recombination have previously been provided [1,2,3,4], our introduction does not contain a thorough, point-by-point referral to the original literature.

HR is also known as general recombination. Initial evidence for the propensity of DNA to undergo HR arose from the early studies of chromosome behavior during meiosis. For example, Tomas Hunt Morgan received the Nobel prize for his experimental research on genetics in drosophila, through which he established the chromosome theory of heredity. At present, homologous DNA recombination is a general term used to include the exchange of information between chromatids: (reciprocal) crossing over, gene conversion, and post-meiotic segregation. Tetrad analysis of all of the products of individual meiotic recombination events revealed that recombinants between distant markers are produced in pairs, with all markers showing normal 4:4 segregation. Such recombination events are called crossovers. Crossover reflects reciprocal exchange between homologous non-sister chromatids. In contrast to crossover, gene conversion reflects the non-reciprocal transfer of genetic information from one duplex to its homolog, with all markers showing 6:2 segregation. In another class of recombination, post-meiotic segregation, a spore is produced that divides to create two genetically different daughter cells. Post-meiotic segregation leads to aberrant 4:4 or 5:3 segregation. Therefore, any molecular model of homologous DNA recombination must reasonably explain the three classes of recombinogenic events described above. It is important to point out that gene conversion is now thought to be a type of non-Mendelian segregation of heterozygous markers near the recombination initiation site [5]. That is to say, it includes both gene conversion and the post-meiotic segregation previously described.

With the continuous expansion of experimental data, a more representative molecular model for DNA meiotic recombination usually requires repeated revisions and upgrades. For example, DNA meiotic recombination has been carried out using the Holliday model (Figure S1A) [6], the Meselson–Radding model [7], the double-strand-break repair (DSBR) model [1], and the synthesis-dependent strand annealing (SDSA) model [8]. Considering that the SDSA is now thought to give rise to most or all NCOs, with the DSBR forming mainly COs, and that SDSA arises from a common precursor, a DNA double-strand break, as the DSBR does, we first focused on the review of the DSBR model, and additionally the Holliday model, which was the first widely accepted molecular explanation for DNA meiotic recombination.

The Holliday model was proposed by Holliday in 1964 [6]. The model describes meiotic recombination that is initiated via single-strand nicks at homologous sites of two homologous chromatids. It postulated a central intermediate containing a Holliday junction (HJ). The HJ is then resolved, giving rise to either crossover or non-crossover recombinants, both of which contain heteroduplex DNA (hDNA) (Figure S1A). Gene conversion and post-meiotic segregation arise from the repair of mismatches on both chromatids in a region of symmetric hDNA (Figure S1B).

In 1976, electron microscope observations of HJs by Potter and Dressler [9] provided direct evidence for HJs in the Holliday model. However, there are still several constraints for this model. First, the accurate occurrence of single-strand nicks at homologous sites of two strands of the same polarity is required for this model, but seems to be very coincidental. Second, according to the Holliday model, mismatches in the heteroduplex should have an equal probability of being corrected in either direction (to wild-type or mutant). However, subsequent experimental data indicate that mismatch correction must use only the invading strand as a template for repair. As a result, Szostak et al. proposed the DSBR model in 1983 (Figure S2A). The elaboration of DNA recombination has been considerably enhanced through studies of this model.

The prominent features of the DSBR model are as follows: (1) meiotic recombination is initiated via the formation of programmed DNA DSBs, and the DSB ends play a prominent role in their repair; (2) the repair of a double-strand gap is completed by two rounds of single-strand repair synthesis; (3) the interacting DNA within joint molecule (JM) intermediates is linked by a double Holliday junction (dHJ) rather than a single HJ; (4) asymmetric regions of hDNA flanking the region of gap repair exist; and (5) the completion of the repair event occurs through the resolution of two Holliday junctions.

The DSBR model can provide the following explanation for the observed properties of meiotic recombination. Two ways can lead to gene conversion (6:2 segregation). When the marked site is within a double-strand gap, a double-strand transfer of information will convert it, and hDNA is not directly involved in this process. In contrast, if the marked site falls within either of the flanking regions of asymmetric hDNA, 6:2 segregation will be caused by mismatch correction. When a mismatch in hDNA remains unrepaired, it leads to post-meiotic segregation [1].

The prediction of a dHJ from this model is supported by electron microscopic observations on DNA from meiotic yeast cells. Bell and Byers (1983) [10] isolated DNA from meiotic cells at the time of recombination, cross-linked the strands to prevent branch migration, cleaved the DNA with a restriction enzyme, and examined the DNA by using an electron microscope. Most molecules joined at homologous sites were joined not by a single HJ, but by a fused region or by two junctions separated by 100–1000 bp [10]. The prediction is further validated by the generation of four-strand intermediates (referred to as JMs) containing two HJs and the identification of these JMs as intermediates in meiotic recombination [11,12,13].

Although the DSBR model has greatly enhanced our understanding of DNA recombination, however, as pointed out, there remain dozens of fundamental questions for which we have no answers and not even very good guesses [2]. For example, with mounting studies, more and more specific constraints are placed on several processes invoked by the model. First, the DSBR model puts forward a specific configuration of hybrid DNA regions beside the initiating DSB site; one of the two recombining chromatids is expected to have a heteroduplex patch to the left of the initiating DSB, while the other recombining chromatid is expected to have a heteroduplex patch to the right. Subsequently, the predicted bi-directional configuration of heteroduplex segments was found to be rare [14,15,16]. Second, JMs have been detected in advance of hDNA [12], which runs counter to the expectation presented in the DSBR model that hDNA is formed early during strand invasion. Third, like the Holliday model, the strand specificity of mismatch repair in the heteroduplex is not completely resolved. Fourth, as has been discussed, increasing data indicate that there should be more pathways which may yet appear [2].

Considering these significant issues and increasing experimental data, we think it is necessary to make revisions to previous models and reanalyze these data to explore structural details and molecular mechanisms of DNA meiotic recombination. This work is a Model and Speculation. We greatly appreciate the contributions of those investigators whose extensive study on DNA recombination has provided the data upon which this study is based.

## 2. Materials and Methods

### 2.1. Literature Search

Pubmed/Medline were searched for relevant articles through 30 June 2013, with a language limitation of English. The search strategy was based on a combination of the key words “DNA Meiotic recombination”, “Crossing Over”, “Holliday junction”, “DNA Replication”, “DNA Damage”, “DNA Repair”, “Gene Conversion”, “Nucleic Acid Heteroduplexes”, and “Genetic”. Reference lists from retrieved documents were also scanned. Two authors independently screened citations and abstracts of each article (Lei Jia and Jingyun Li).

### 2.2. Selection Criteria

If they met the following selection criteria, studies should be included: (1) studies published in English; and (2) studies focusing on molecular model of DNA meiotic recombination, transpositional recombination, and site-specific recombination.

### 2.3. Data Collection

Two authors extracted data independently and recorded the following for each publication: published year, details of study design, experimental results, and representative viewpoints. Where eligible, articles with sufficient data were included.

### 2.4. Study Quality

The two authors also assessed study quality based on the following criteria: (1) the institute where the studies were performed; (2) the citation number; and (3) the journals of publication.

### 2.5. Model Revisions and Upgrades

A biophysical modeling method based on objective physical and biological rules was used to make revisions and upgrades to previous models. Finally, a new model was established.

### 2.6. Data Reanalysis

The collected data was reanalyzed based on the new model in detail to obtain further interpretations.

## 3. Results

### 3.1. Search Results

DNA recombination-related records were obtained by searching literature databases such as Pubmed/Medline. These records were further screened by reading their titles and abstracts, and then some records were excluded for unrelated studies. The full texts of the remaining studies were read carefully, and some of them were further excluded as they did not focus mainly on the molecular model of DNA meiotic recombination. Eventually, 58 studies met the predefined selection criteria and study quality.

### 3.2. Data Collection Summary

We obtained all data directly from the included studies. Table 1 provides details such as references, data/result(s), and representative viewpoints of some key selected studies. In total, five key issues were extracted: (1) sequence of the appearance of hDNA, JM, and recombination products; (2) accurate configuration of hDNA; (3) strand specificity during mismatch repair of hDNA; (4) accurate number of HJs involved in recombination; and (5) actual mechanism of site-specific recombination—break/join or copy choice? Other selected data are embodied within the main text.

### 3.3. Model Revisions and Upgrades Results

Our model is designated the intertwinement model. This mechanism was originally proposed in our other study to explain HIV-1 recombination generated during reverse transcription [17]. A branched structure (BS), in which two single-stranded genomic RNAs are topologically cross-linked to form a junction, can mediate the copy choice of reverse transcriptase between two templates, thus leading to genetic recombination. The key similarities between HIV-1 reverse transcription and DNA replication (i.e., both requiring nucleic acid strand synthesis based on another nucleic acid strand) led us to consider whether DNA recombination might also be initiated in a similar manner.

Meiosis is a cell division program in which a single round of DNA replication is followed by two consecutive rounds of chromosome segregation, thereby allowing the formation of haploid gametes from diploid germ cells [18,19]. It has been shown, first in a mouse, that DNA synthesis occurring during pachytene is involved in meiotic recombination [20,21]. Since DNA synthesis occurs in a liquid environment, relative motion between DNA strands may be generated as a result of liquid streaming, diffusion, or supercoiling. Typically, relative motion will favor strand intertwinement. When the intertwinement occurs between single-stranded DNAs during synthesis, it causes a BS, which, in turn, initiates recombination via mediating copy choice [17].

**Figure 1:**
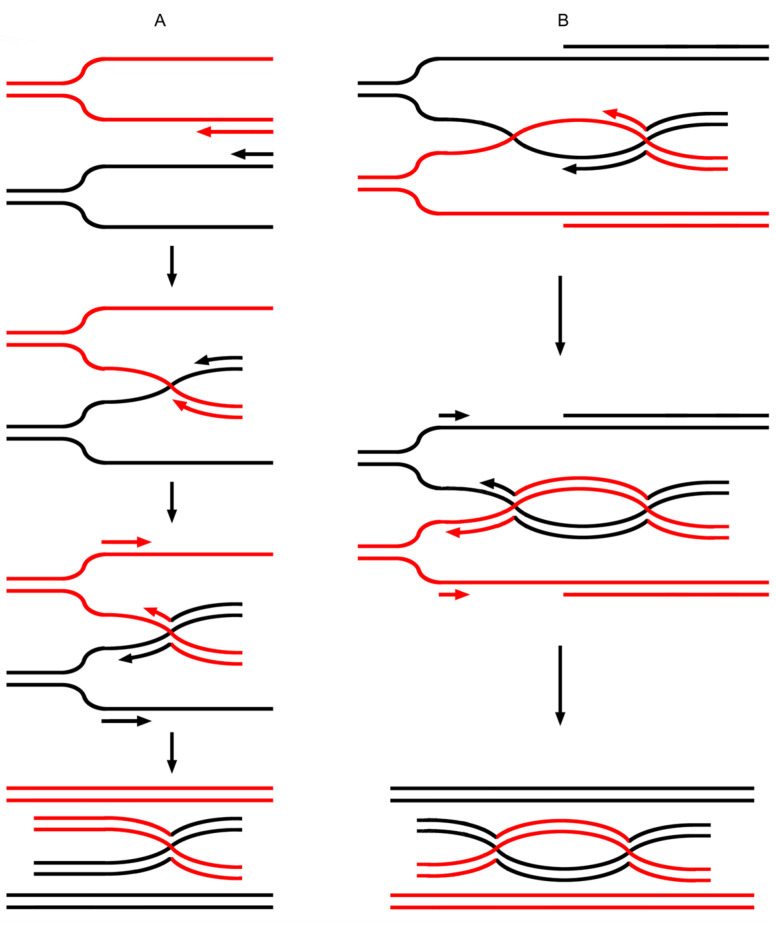
The BS-based copy choice results in HJ formation [17]. (**A**) A BS arises between two single-stranded DNAs of identical polarity during DNA synthesis. As the BS is consecutively encountered by polymerases that engage maternal and paternal DNA separately, it mediates two rounds of copy choice, with the end result being a single HJ. (**B**) A second BS forms downstream of the initial HJ. When the maternal and paternal polymerases each encounter this second BS, they undergo the same copy-choice process as they did at the first BS, ultimately resulting in the formation of a dHJ. The red and black arrows in the strands indicate the direction of synthesis extension.

In the current model, DNA polymerase will undergo a “climb process” as it encounters a BS. While different results are generated under different conditions, in the present study, two of these potential results are shown in Figure S3. The first scene shown in Figure S3A would lead to copy choice. Two rounds of such copy choice at a BS would result in a structure that is exactly an HJ (Figure 1A).

In contrast to the DSBR model, where a dHJ is expected to arise from a double-strand break (DSB), capture of the end, DNA synthesis, and ligation, a dHJ can form via a double-BS within the current model, as shown in Figure 1B. The process described above provides a much simpler method of HJ formation, i.e., an HJ can be generated from copy choice based on a BS during DNA synthesis. Next, we addressed the nature of the later stages of recombination (i.e., various recombination events).

**Figure 2:**
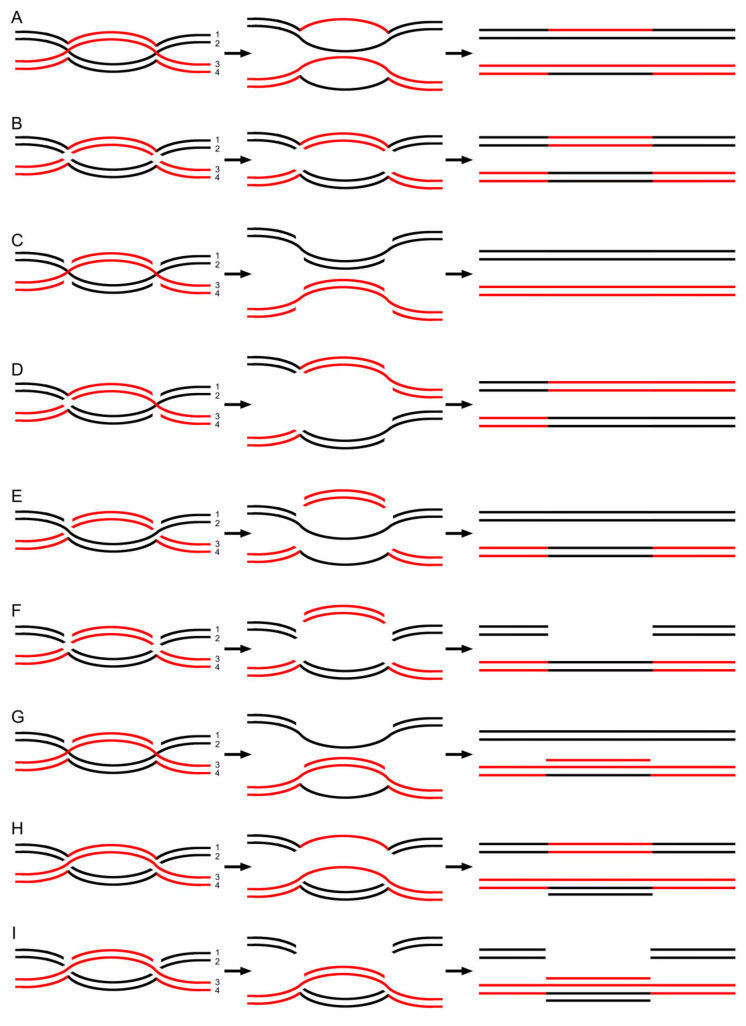
A range of resolution patterns is capable of resulting in distinct recombination events [17]. (**A**) Prior to the resection of both HJs, the Meiosis I spindles start pulling maternal and paternal chromosomes toward opposite directions. Topologically, this leads to the resolution of the dHJ and the generation of two symmetric heteroduplexes. Following this, post-meiotic segregation takes place, manifesting as an aberrant 4:4 segregation pattern. (**B**) When the two junctions are resolved by cleaving their inner, crossed strands, it leads to the crossover of genes between the two HJs. (**C**) Cutting the outer, non-crossed strands to resolve the two junctions has no impact on the products after repair synthesis. (**D**) In contrast to (**B**), opposite-sense cleavage—for instance, cutting the crossed strands in the left-hand HJ and the non-crossed strands in the right-hand HJ—produces crossover that contains upstream regions. (**E**) In both HJs, cuts are introduced on strand 1 and strand 3. Following the pulling force exerted by the meiosis I spindle, two duplexes form—each containing a single-strand gap. These two gaps are then repaired through repair synthesis, ultimately resulting in gene conversion (manifested as a 6:2 segregation pattern). (**F**) In both HJs, cuts are introduced on strands 1, 2, and 3. Following the pulling force and repair synthesis, the information from the black chromatid is transferred to the homologous region of the red one, while the original genetic information on the black chromatid is deleted, resulting in gene conversion of deletion. (**G**) In both HJs, cuts are introduced on strand 1. The resolution produces one duplex with a single-strand gap and another duplex containing a three-stranded helix. Inside this three-stranded region, a duplex formed by strand 1 and strand 3 initially appears; subsequently, this duplex interacts with strand 4, leading to the formation of three-stranded helical DNA. After mitosis, the three-stranded helical DNA generates post-meiotic segregation, which presents as a normal 5:3 segregation pattern. (**H**) Unlike (**G**), cuts are introduced on strand 2 in both HJs. Post-meiotic segregation occurs, yet the resulting products exhibit an aberrant 5:3 segregation pattern. (**I**) When strands 1 and 2 in both HJs are cleaved, the genetic information on the black chromatid is deleted and transferred to the red chromatid. Subsequently, a four-stranded helical DNA—composed of two duplexes—emerges. Following mitosis, this four-stranded helical DNA produces post-meiotic segregation.

The previous DSBR model concentrates on the two resolutions of each HJ, that is, cutting either the originally crossed strands or non-crossed strands, as a mechanism to explain DNA meiotic recombination. The resolution pattern was originally proposed by Holliday and is subsequently implicit in all analyses of meiotic recombination since that time [6]. The pattern would lead to four possible resolutions of each dHJ. An HJ, however, consists of four component single strands. When an endonuclease randomly resects an HJ through digestion, the resolution process ought to exhibit greater variability. For instance, endonucleases may digest any one, two, three, all four, or none of these strands. Consequently, each HJ should have 16 potential resolution outcomes, and each dHJ would therefore have 256 possible resolutions. To account for observations such as crossing over, gene conversion, and post-meiotic segregation, nine specific resolution patterns are thoroughly described in Figure 2 [17].

From the intertwinement model described above, we can conclude that crossover arises from cutting the crossed strands of each HJ (Figure 2B), or opposite-sense cutting (Figure 2D). The process in Figure 2E would result in gene conversion and the events illustrated in Figure 2F would lead to gene conversion of deletion. Post-meiotic segregation appears when any of the events described in Figure 2A,G–I occur. In light of the current model, post-meiotic segregation can arise at least in two ways. First, it is a product of hDNA without mismatch repairing. Second, it can arise from a three-stranded DNA helix.

### 3.4. Data Reanalysis: The Intertwinement Model Embodies the Particular Characteristics of the SDSA Model

Allers and Lichten noted differences in the properties and the timing of appearance of COs and NCOs; this led them to propose that a major portion of NCOs arise not from a mechanism that involves a ligated dHJ, but instead from synthesis-dependent strand annealing (SDSA) [8]. Homology-mediated repair of double-strand breaks (DSBs) does not need the formation and resolution of ligated HJs in the SDSA (Figure S2B). The SDSA model had its simplest version of accounts for NCO products and obtained essential support. One key aspect of this support was that a recipient chromatid has the ability to collect sequences from donor loci [22,23]. The second one is the demonstration of a genetic signature of less-stable recombination intermediates than HJs needed to make crossovers [5]. However, it is important to note that the intertwinement model has also embodied the particular characteristics of the SDSA model. First, the current model can easily explain differences in the timing of appearance of COs and NCOs. As shown in Figure 2, besides the different recombinogenic products, those various resolution patterns would also lead to differences in the time of products’ appearance. For example, there is no strand cut in Figure 2A, and thus NCO products from the resolution would form earlier than others shown in Figure 2. There is only one strand cut at each HJ in Figure 2G,H; therefore, their NCO products would be the second-earlier. Successively, the products in Figure 2B–E,I would be the third-earlier. Products in Figure 2F would be last, as there are three strand-cuts at each HJ. Second, copy choice is obviously a mechanism in which a recipient DNA could collect sequences from the donor loci. Third, we would like to stress that what we want to express most via this work is a concept. The core of the concept is that during nuclear acid strand synthesis based on another nuclear acid strand (as observed during replication, transcription, and reverse transcription), a BS can be generated, and it could affect the forward path of the polymerase, leading to copy choice. It is this critical event during synthesis that would lead to genetic recombination. Therefore, recombinogenic events should not be restricted to an HJ because copy choice can certainly lead to other types of junctions. For example, a round of copy choice at each BS will result in a simpler and less stable three-stranded junction, as shown in Figure S4. After nucleic acid breakage and rejoining, recombination might also appear.

### 3.5. Data Reanalysis: hHDNA Can Also Arise from JM Resolution Rather than Being Followed by a JM

According to the DSBR model, hDNA forms early during strand invasion and is followed by a JM. Finally, crossover products are generated via JM resolution. However, two data sets conflict with this proposed mechanism. First, JMs have been detected in advance of hDNA [12]. Second, physical analysis has shown that experimentally detectable hDNA emerges just prior to, or concomitantly with, the appearance of mature recombination products (summarized in [13]). In the intertwinement model, JM first forms due to copy choice; then, after topological resolution, hDNA arises (Figure 2A). Because hDNA is no longer a precursor of segregations but a product of JM resolution, it is much closer to crossover and non-crossover products. Then, the inexplicable inconsistency receives a simple interpretation.

Additionally, the DSBR model predicts a specific asymmetric configuration of hDNA regions relative to the site of the initiating DSB (Figure S2A). However, the expected bi-directional configuration of heteroduplex segments was found to be rare [14,15,16]. Instead, other unexpected configurations were found. For example, in one recombinant class, hDNA was found only on one side of the initiating DSB. In a second recombinant class, two tracts of heteroduplex were found, both on the same chromatid [15,24]. The DSBR model cannot account for this type of recombinant. In contrast, the unexpected observations exactly correspond to a natural consequence in the intertwinement model (see hDNA configuration in Figure 2).

### 3.6. Data Reanalysis: Strand Specificity of hDNA Mismatch Repair Seems to Be an Illusion and Copy Choice Is More Likely to Be the Actual State

Mismatch correction of heteroduplexes plays a key role in segregations according to the DSBR model. HDNA has an equal probability of being corrected in either direction (to wild-type or mutant). If an hDNA mismatch is not repaired, post-meiotic segregations occur. However, experiments by Savage and Hastings with the *his1* locus and by Fogel et al. with the *arg4* locus suggest that mismatch correction must use only the invading strand as a template for repair, indicating that there is an apparent strand specificity of mismatch repair (for review, see [1]). Copy choice is obviously a simpler version of accounts for this mystery. As shown in Figure 1, after BS-based copy choice, the synthesis would be elongated along the new template (homologous DNA comes to ligation via invasion, according to previous models (Figure S2)). As a consequence, the final result naturally represents the genotype of the acceptor strand (“the invading strand”).

### 3.7. Data Reanalysis: Parity in Resolution Patterns of a dHJ Would Lead to Parity of Gene Conversion

**Figure 3:**
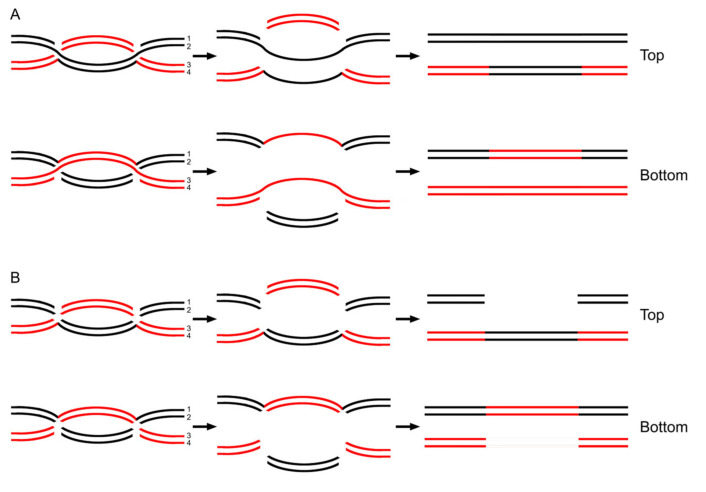
Parity of both gene conversion and gene conversion of deletion. (**A**) When cuts are made on strand 1 and strand 3 in both HJs, after the pull and repair synthesis, the product would show gene conversion of information transferred from the black chromatid to homologous regions of the red one (Top). Symmetrically, when cuts are made on strand 2 and strand 4, the product will show gene conversion of information transferred from the red chromatid to the black one (bottom). (**B**) When cuts are made on strand 1, strand 2, and strand 3 in both HJs, after the pull and repair synthesis, the product would show gene conversion of deletion. The information is transferred from the black chromatid to homologous regions of the red one. Simultaneously, the original information in the black chromatid is deleted (Top). Symmetrically, when cuts are made on strand 2, strand 3, and strand 4, the information would be transferred from the red chromatid to the black one. Synchronously, the original information in the red chromatid is deleted (bottom).

It does not escape our notice that a dHJ is a symmetrical structure. And, therefore, this would lead to parity in resolution patterns. This parity in resolutions would reflect an equal probability of product types (Figure 3). Thus, a novel and simple interpretation can be provided for the following phenotypes. It has long been known that all classes of mutations in yeast show parity [25,26]. This means that gene conversion favors neither the wild-type nor the mutant allele. In addition, gene conversion of large deletions in yeast also shows parity [27,28].

### 3.8. Data Reanalysis: The Cooperation of Multiple HJs Would Readily Generate High Correlation Between Gene Conversion and Crossover

**Figure 4:**
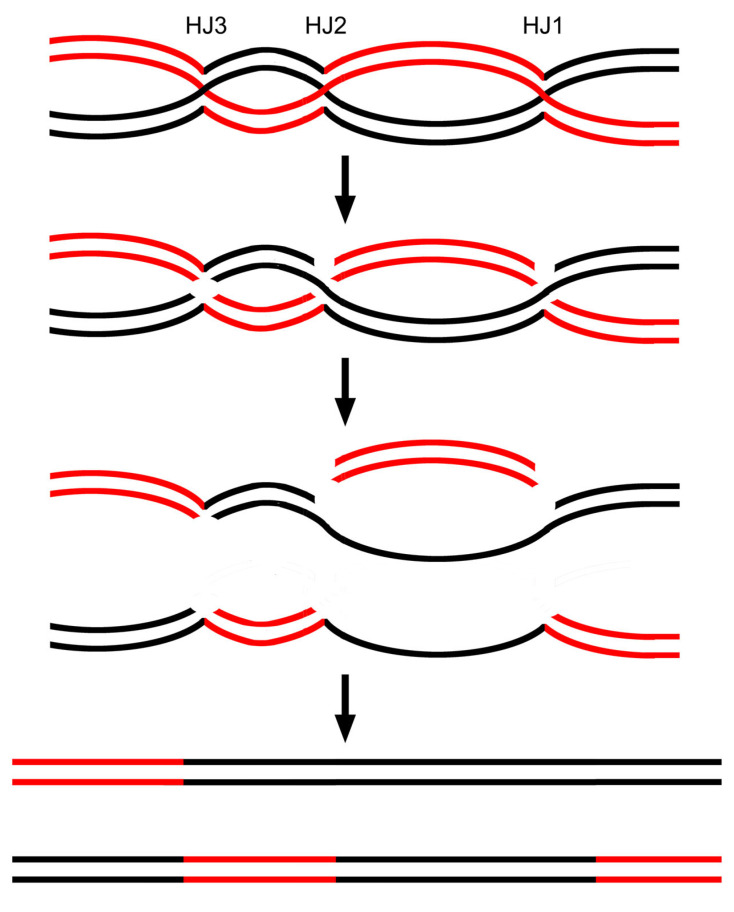
A single HJ and a dHJ would readily lead to high association between gene conversion and crossover. A third Holliday junction (HJ3) forms downstream of the dHJ (HJ1 and HJ2). When the resolution of HJ1 and HJ2 cooperatively produces gene conversion and the resolution of HJ3 generates crossover, the final result would display a crossover that lies on the left-hand side of the gene conversion site.

Through extensive tetrad analysis in yeast and other lower-order fungi, researchers have observed that any type of aberrant segregation is linked to a high likelihood of reciprocal exchange occurring nearby. Specifically, when a site displays conversion or post-meiotic segregation, properly segregating sites on either side also show crossing-over at a frequency of up to 50%—even when these markers are tightly linked (summarized in Ref. [1]). Within the DSBR model, conversion and post-meiotic segregation both originate from the regions between the two HJs. Because the two HJs to resolve flank two sides of gene conversion, the resulting crossover may genetically lie on either side of the conversion site. The concern is that the bi-directional configuration of heteroduplex segments postulated in the DSBR model is rare [14,15,16]. Therefore, the DSBR is now thought to form mainly COs and the SDSA model was proposed to specially account for gene conversion [5,8]. In light of the current model, first, both gene conversion and post-meiotic segregation can be generated from the resolution of a dHJ, the same as crossover (Figure 2). Second, it should be noted that here there is no restriction in the number of HJs in a round of DNA synthesis. For example, as shown in Figure 4, a third HJ involving the same two crossing strands can be generated downstream of the dHJ, thus forming a three-HJ JM. The combination of HJ-1 and HJ-2 resolution can result in gene conversion and HJ-3 resolution can lead to crossover, thus displaying a crossover near a conversion. Due to equal chances, there may be a third HJ on either side, yielding crossover lying on either side, as observed by Fogel et al. [26].

### 3.9. Data Reanalysis: Transpositional Recombination and Site-Specific Recombination May Have a Common Pathway to Meiotic Recombination

**Figure 5:**
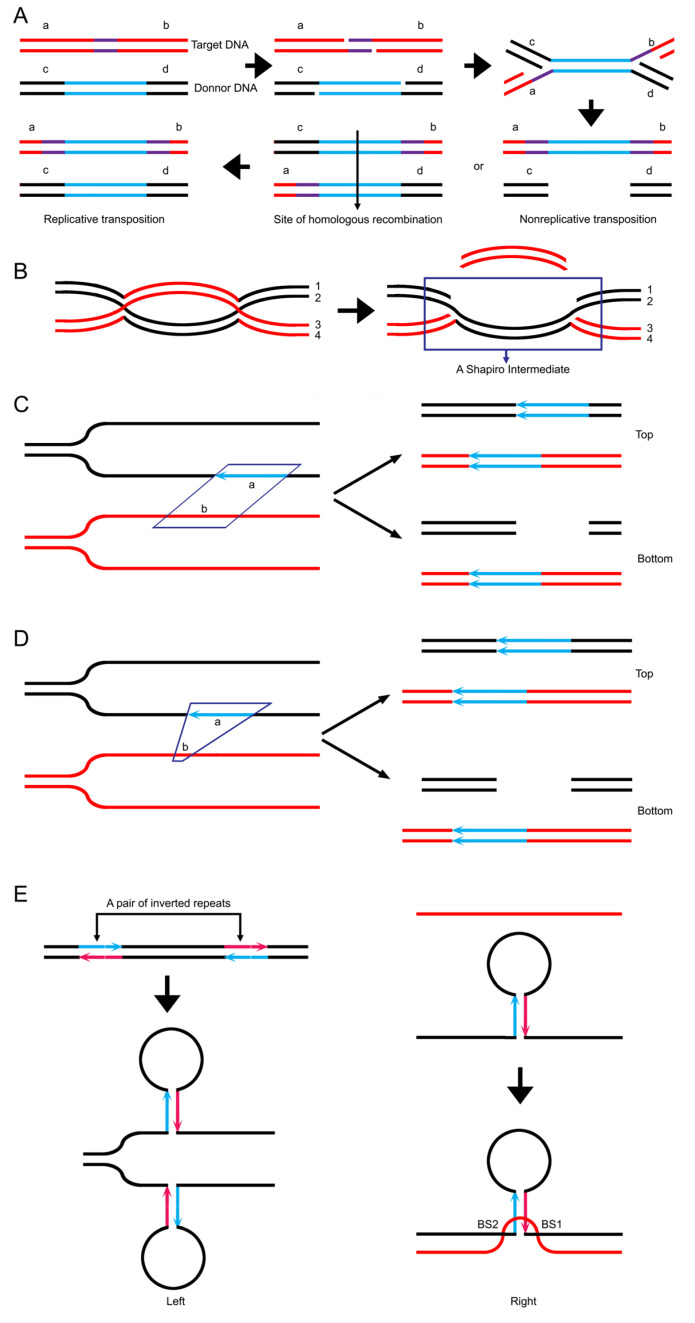
Comparison of the Shapiro model and the intertwinement model. (**A**) The Shapiro model. The donor molecule is cleaved on either strand at the extremity of the transposable element. The target molecule is cleaved to yield 5 or 9 bp cohesive ends. The donor and target strands are ligated to generate an χ-shaped structure called a Shapiro intermediate. Subsequently, after breakage and rejoining, non-replicative transposition occurs. After release, repair replication and homologous recombination, replicative transposition occurs. The purple region indicates the oligonucleotide target sequence. The blue region indicates the transposable element. The letters a, b, c, and d in the duplex arms flanking the transposable elements and target oligonucleotide serve to indicate the genetic structure of the various duplex products. (**B**) Resolution of a dHJ would also result in a structure that is exactly the same as the Shapiro intermediate. The pattern of resolution here is the same as the one shown in Figure 2E. (**C**) Intertwinement occurs at non-colinear positions. The lengths of two fragments of participants are equal, i.e., fragment a = fragment b. The resolution as shown in Figure 2E leads to transpositional gene conversion, similar to replicative transposition (top). The resolution shown in Figure 2F leads to transpositional gene conversion of deletion, similar to non-replicative transposition (bottom). (**D**) The length of two participating fragments varies, where fragment a > fragment b. The resolution as shown in Figure 2E leads to transpositional gene conversion of insertion, i.e., replicative transposition (top), while the resolution as shown in Figure 2F leads to transpositional gene conversion of insertion and deletion, i.e., non-replicative transposition (bottom). (**E**) During DNA replication, the duplex is released into single strands. Thereafter, inverted repeats within a single strand can readily induce a hairpin (left). When the hairpin participates in the intertwinement with a single strand, the structure involving a pair of BSs (right) could appear. After recombination based on the pair of BSs, the whole hairpin would be moved as an independent element.

A transposon can independently transpose from one DNA site to another in an event called transpositional recombination. The Shapiro model proposed by Dr. J. Shapiro explains the molecular mechanism underlying this event [3]. This model postulates a Shapiro intermediate and explains both replicative transposition and non-replicative transposition (Figure 5A). However, it should be noted that the particular Shapiro intermediate is not a unique structure. It can result in the intertwinement model more easily (Figure 5B). Therefore, it can be concluded that in addition to the function in interpreting HR, our model indicates that there may be a common foundation for transpositional recombination. With respect to the mechanism by which a gene can be translocated via recombination, our model provides the following interpretations: intertwinement can occur not only at colinear positions (i.e., homologous recombination), but also at non-colinear positions. Therefore, after copy choice based on an irregular double BSs, an asymmetric dHJ would appear. The resolution of this dHJ would lead to transpositional recombination (Figure 5C,D). Then why can a transposable element be translocated as an independent part during the process? We think the pair of inverted repeats flanking a transposon should play a key role. Inverted repeats would readily induce the formation of a hairpin within a single strand during DNA replication. When the hairpin participates in intertwinement, the resulting structure containing a pair of BS shown in Figure 5E could appear. When similar procedures to Figure 2 occur, the whole hairpin can be moved as an independent component from the donor to the acceptor. It is important to point out that inverted repeats inducing a hairpin structure were once proposed by d’Alencon et al. in their research concerning nearly precise excision of a transposon to study illegitimate DNA recombination between short direct repeats [29].

**Figure 6:**
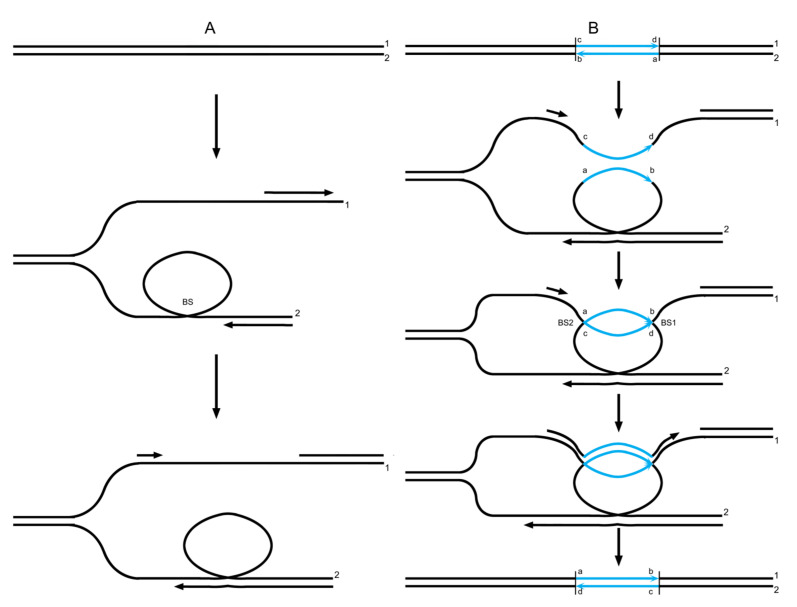
The interpretation for site-specific recombination by the intertwinement model. (**A**) Gene deletion: During DNA synthesis, an intra-strand BS forms via intra-strand intertwinement (within strand 2). After copy choice, DNA polymerase directly reaches the left part of the BS (sequence homology flanking the BS site can induce the process). As a result of not being a template, the loop region is deleted. (**B**) Gene inversion: The loop could intertwine with strand 1, resulting in a pair of BSs. After copy choice, resolution, and repair synthesis, as shown in Figure 2, the blue region is inverted in the progeny. It should be pointed out that the illustration here only describes two of many possibilities. Numbers 1&2 represent the two single strands in the double-stranded DNA respectively. Segment ab represents a marker region within strand 2. Segment cd represents a marker region within strand 1. The black arrows in the strand indicate the direction of synthesis extension.

Another type of non-HR is site-specific recombination, which is summarized in a previous reference [4]. Site-specific recombination refers to recombination that takes place between specific DNA sequences. This process was first identified during the genetic research on the λ phage. For sites located on the same chromosome, the current understanding indicates that the outcome of recombination is determined by their relative orientation. Specifically, recombination between sites arranged in a head-to-tail orientation leads to excision, while recombination between inverted (head-to-head) sites results in inversion. According to the novel model, intertwinement can occur not only between single-stranded DNAs from two duplexes, but also between two strands of one duplex. In addition, such intertwinement is available between regions on one single-stranded DNA, resulting in intra-strand intertwinement. The latter two cases would also naturally generate a gene deletion (Figure 6A) or inversion (Figure 6B).

Previous studies focused on recombination events that involved short direct repeated sequences. These events belong to a class of illegitimate recombination, which takes place between sequences with little to no homology [30], and have been documented in prokaryotes [31,32,33], along with both lower and higher eukaryotes, including humans [34,35,36,37,38]. Several strands of genetic evidence suggest that recombination of short direct repeats might result from DNA replication errors—specifically, the tip of a growing DNA chain could slip between repeats and be utilized as a primer to continue DNA synthesis [31,39]. This type of model was first proposed to account for frame-shift mutations [40] and is conceptually linked to copy choice recombination [41]. Of note, d’Alencon et al. employed the nearly precise excision of a Tn10-related transposon from an *Escherichia coli* plasmid as a model to investigate illegitimate DNA recombination between short direct repeats [29]. They demonstrated that nearly precise excision was significantly stimulated by rolling circle replication, and this process did not involve the transfer of DNA from the parental molecule to the recombinant molecule. Both findings clearly support a copy-choice mechanism—rather than a break/join mechanism—for recombination between short direct repeats. Additionally, we are pleased to note that the reanalysis aligns well with these experimental results. 

**Table 1:** Previously published studies and results related to DNA meiotic recombination.

Reference(s)	Result(s)/Data	Representative Viewpoint(s)
[1]	hDNA forms early during strand invasion and is followed by a JM. Finally, crossover products are generated via JM resolution.	hDNA > JM > products
[12], See summary in [13]	JMs have been detected in advance of hDNA. Experimentally detectable hDNA emerges just prior to, or concomitantly with, the appearance of mature recombination products	JM > hDNA ≈ products
[1]	A specific asymmetric configuration of hDNA regions—relative to the site where the initiating DSB occurs—is what the DSBR model predicts (Figure S2A).	
[14,15,16]	The bi-directional configuration of heteroduplex segments, which was predicted by the DSBR model, was rarely observed.	
[1,6]	hDNA has an equal probability of being corrected in either direction (to wild-type or mutant).	No strand specificity during mismatch repair.
See summary in [1]	Experiments by Savage and Hastings with the *his1* locus and by Fogel et al. with *arg4* locus suggest that when carrying out repair, the invading strand is the only template that mismatch correction is permitted to use.	There is an apparent strand specificity during mismatch repair.
See summary in [4]	Excision originates from exchange between sites in a head-to-tail orientation, while inversion arises from recombination between inverted (head-to-head) sites.	The outcome is determined by a break/join mechanism.
[31,39]	Recombination of short direct repeats might result from DNA replication errors—specifically, the tip of a growing DNA chain could slip between repeats and be utilized as a primer to continue DNA synthesis.	Strongly support that recombination between short direct repeats operates via a copy-choice mechanism, rather than a break/join mechanism.
[29]	Nearly precise excision was significantly stimulated by rolling circle replication, and this process did not involve the transfer of DNA from the parental molecule to the recombinant molecule.	The same as above.

## 4. Discussion

The movement of transposable elements (TEs) within the genome, including endogenous retroviral (ERV) elements, exerts a powerful and continuous shaping influence [42,43,44,45,46]. The current work performed a larger scale of reanalysis of experimental data collected from the past decades. Simultaneously, a revision to previous recombination models was made. The results indicate that both the revisions and reanalysis are very necessary. In fact, direct evidence for the intertwinement model also exists. For example, this model suggests that DNA recombination occurs during DNA synthesis. This notion is supported by the fact that when performing transformation with closed circular plasmid DNA, more transformants can be recovered by using plasmids capable of autonomous replication [47,48]. This model also indicates that a dHJ can be resolved not via a junction-specific nuclease, but topologically (Figure 2A). This notion can be strengthened by experimental data from Thaler et al., who show that a dHJ can potentially be converted into a pair of mature, non-crossover recombination products through topoisomerase and without the action of a junction-specific nuclease (e.g., [49]). Moreover, as shown in Figure 2G,H, the model proposes the generation of a three-helical DNA structure composed of a single strand and a duplex. The rationality of such a DNA class is supported by previous work, where this DNA type was discovered and has been termed “triplex DNA” [50,51,52]. Taken together, these data provide direct support for the intertwinement model.

In summary, the significant features of our DNA recombination model are that (1) meiotic recombination can be initiated by copy choice; (2) it occurs with the direct involvement of a BS; (3) an HJ can result from BS-based copy choice of DNA polymerase; (4) there are more patterns of HJ resolution than those recognized previously, and the three recombinogenic events can arise from these different resolutions; and (5) hDNA can be a result of HJ resolution.

We hope copy choice initiating meiotic recombination will be one of the concepts that will be explored further, although clearly it should not be the only concept. Importantly, it should be noted that the concept of copy choice initiating meiotic recombination is not unique. Belling first proposed the theory of copy choice in the early 1900s; however, he withdrew the hypothesis in 1933. In 1946, Alfred D. Hershey and Max Delbrück independently discovered the genetic recombination in bacteriophages, demonstrating that viral genes can exchange and generate new types of viruses. Then, subsequently, Hershey discovered that recombinants generated from phage hybridization are sometimes dissymmetrical. To explain this observation, he accepted the suggestion by Sturtevant, and proposed that the recombination of the phage is not simply via breakage and rejoining of genetic material, but via the copy choice during replication. However, a subsequent experiment brought a lethal shock to this popular model of the time. According to Hershey, a whole chromatid is regarded as a template, and a new chromatid shows elongation along the whole chromatid, meaning that this is a conservative replication. In 1958, Meselson and Stahl first demonstrated that DNA replication is semiconservative by using ^15^N-labeled *Escherichia coli* DNA [53]. In the present study, we propose a mechanism that resolved the deficits of Hershey’s model. Specifically, we suggest that the template is not a whole chromatid but a single-stranded DNA.

Currently, the mobile elements, PRDM9, HJ and other recombination hotspots have been indicated to play roles [54,55,56,57,58]. Proper and specific biochemical experiments are required to further reconstruct the detailed mechanism described here. In addition, the intertwinement model proposes another source of gene mutagenesis. As shown in Figure S3B, a very brief synthesis of several or even a base on the new template might result in mutations. Further experiments are necessary to validate this concept.

In conclusion, novel interpretations would be critical to the understanding of the mechanisms of DNA recombination, as well as its role in DNA repair. Additionally, the work could have implications for how the field views the importance of factors such as Spo11 or the mechanisms that drive meiotic pairing.

Without being restricted to meiosis, the recombination events shown in Figure 6 might also occur during mitosis and result in mitotic recombination. In light of the current model, intertwinement between nuclear acid strands is an objective physical rule. Therefore, one can assume that such intertwinement, as well as the resulting recombination event between DNAs of different species, or even between DNA and RNA, should not be an exception. Thus, some other unusual recombination events, such as DNA with RNA, could produce a more reasonable explanation.

## Supplementary Materials

The following supporting information can be downloaded at https://www.mdpi.com/article/10.3390/ijms26199464/s1.
