# Supplementary material for: Meiotic Recombination May Be Initiated by Copy Choice During DNA Synthesis Rather than Break/Join Mechanism
Source: Int J Mol Sci. 2025 Sep 27;26(19):9464. doi: 10.3390/ijms26199464 (PMC12524634; doi:10.3390/ijms26199464)
Supplement: Supplementary file 1 [file ijms-26-09464-s001.zip › ijms-3567599-supplementary.pdf]

Supplementary Figure Legends:

Supplementary Figure S1 The Holliday model.

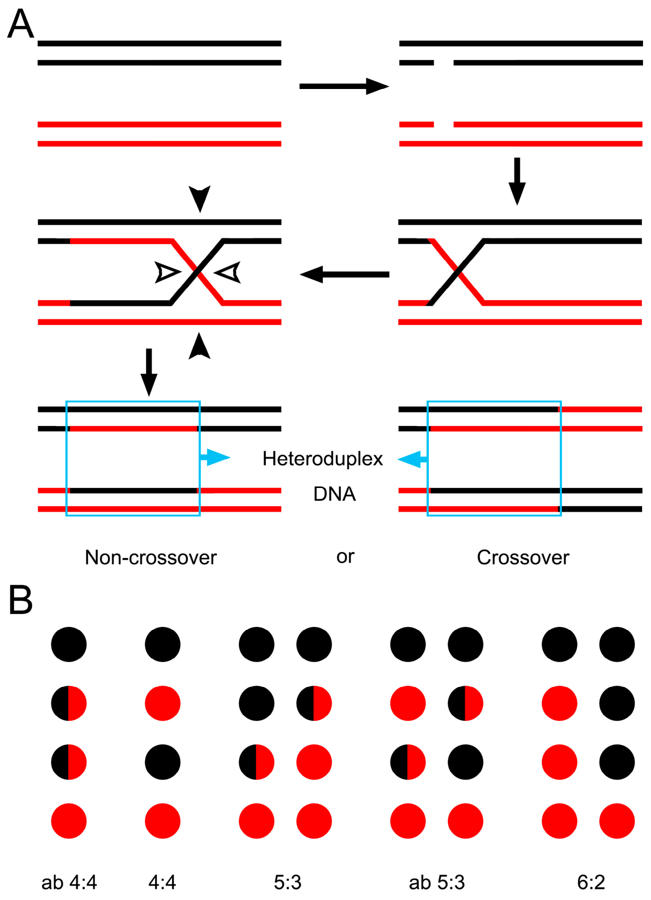

(A) Strands of the same polarity are nicked at homologous sites and the nicked strands are exchanged on one side of the nick, resulting in symmetric heteroduplex DNA and a HJ. The HJ is then resolved by cutting the inner, crossed strands (open arrows), in which case non-crossover occurs, or by cutting the outer, non-crossed strands (filled arrows), in which case crossover occurs.

(B) The genetic results when a single heterozygous site is involved in symmetrical hDNA generated by the Holliday model. When a marked site (solid circles and half circles) falls within the hDNA, mismatches are created and aberrant segregation can occur. If neither mismatch is corrected, the outcome is aberrant 4:4 (ab4:4) segregation (post-meiotic segregation). If the mismatch on the top DNA is corrected from black to red, while mismatch on the low DNA is corrected from red to black, normal 4:4 occurs (crossover). If the

mismatch on one DNA duplex is corrected, normal 5:3 or aberrant 5:3 (ab5:3) segregation occurs (post-meiotic segregation). If the mismatches on both DNA duplexes are corrected in the same direction (e.g., black to red), 6:2 segregation results (gene conversion).

Supplementary Figure S2 The DSBR model and the SDSA model.

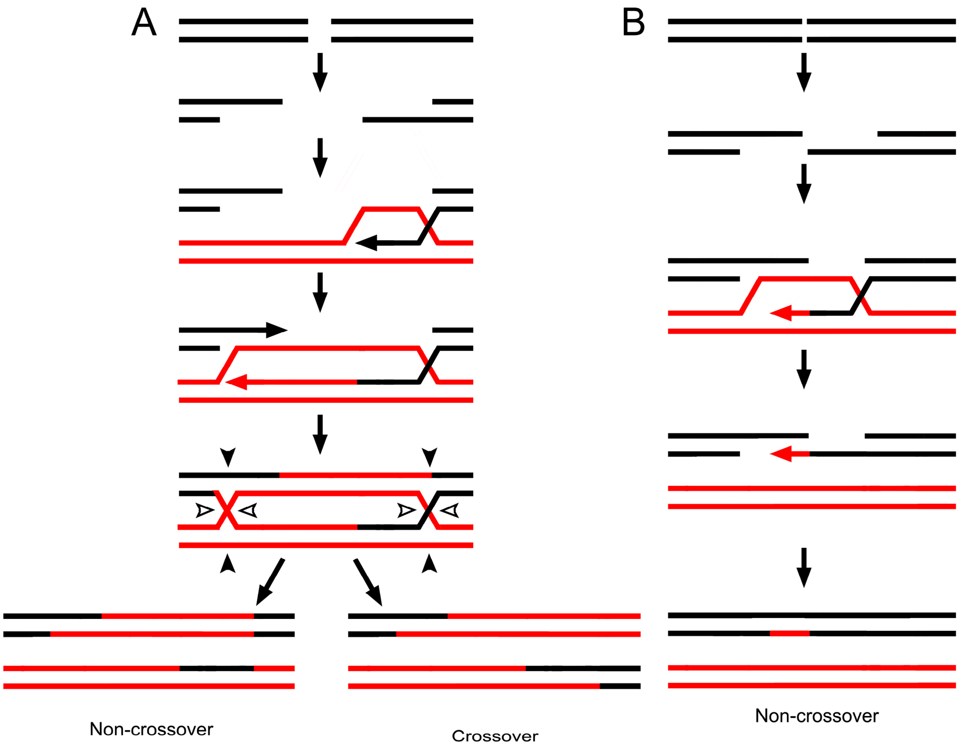

(A) The DSBR model. A double-strand cut is made in one duplex, and a gap flanked by 3' single strands is formed via exonucleases. One 3' end invades a homologous duplex, displacing a D loop. The D loop is enlarged by repair synthesis until the other 3' end can anneal to complementary single-strand sequences. Repair synthesis from the second 3' end completes the process of gap repair, and branch migration results in the formation of a dHJ intermediate with hDNA flanking the DSB site. This intermediate is resolved by cutting the outer strands (filled arrows) or inner strands (open arrows) of each junction. Two of the four possible resolutions are shown here: opposite-sense cutting generates crossovers, and same-sense cutting generates non-crossovers. Both products contain hDNA.

(B) The SDSA model. Ends are resected, and one DSB end forms a D-loop with its homolog and is then extended by DNA synthesis, as in the DSBR model. The newly synthesized DNA strand is displaced and anneals with the other DSB end. Break repair is completed by DNA synthesis and ligation. In this mechanism, hDNA arises at the time of resolution, and only noncrossover products are formed.

Supplementary Figure S3 Two of potential results when DNA polymerase encounters a BS during synthesis.

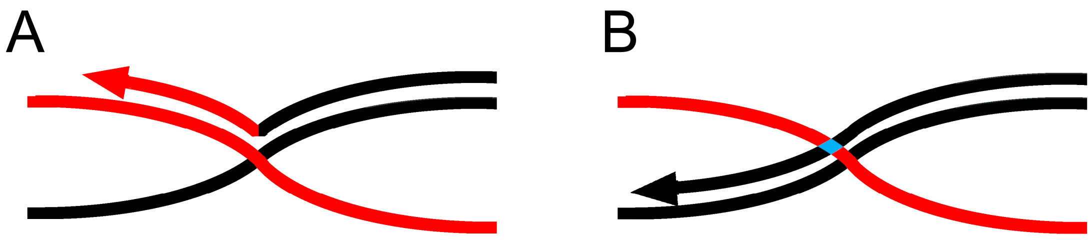

(A) DNA polymerase encounters a BS and pauses. Then, it "climbs up" the acceptor template, and resumes the following synthesis along the new template, resulting in successful copy choice and yielding the recombination junction.

(B) When DNA polymerase encounters a BS, it is able to "climb up" the acceptor template, perform synthesis along the new template, then "climb down" back to the donor template and continue to elongate, resulting in a braid-like intermediate.

Supplementary Figure S4 A less-stable intermediate and noncrossover recombinant from it.

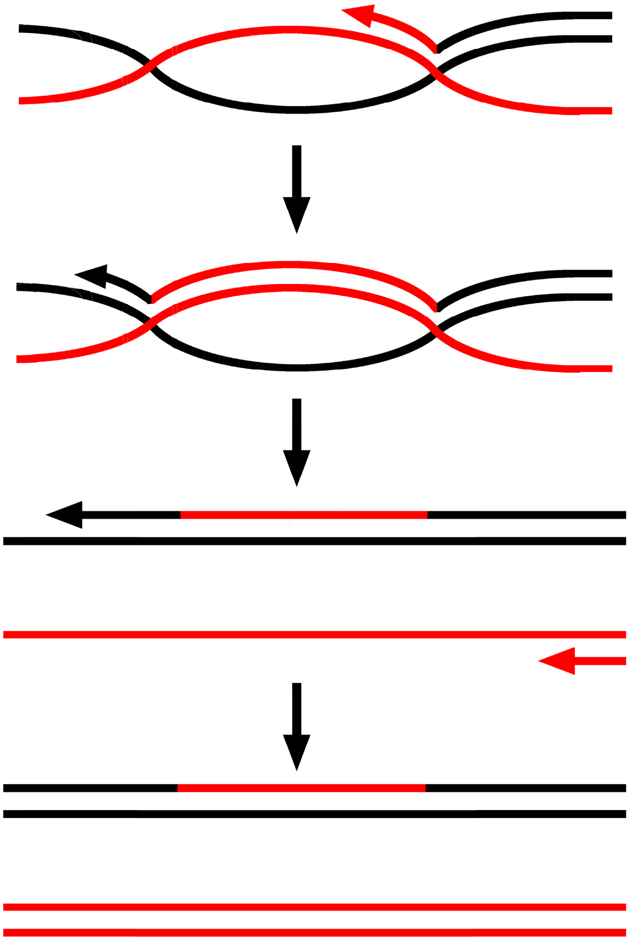

At each BS, only one round of copy choice occurs. Thus, a simpler and less-stable junction than a HJ is generated. The acceptor template is released before any stands are resected. The intermediate would be resolved more easily than a dHJ. After synthesis, noncrossover products are generated. Other than the initiating way, the illustration presents a procedure that is very similar to that in the SDSA model (Figure S2B).
